# Eye movements reflect expertise development in hybrid search

**DOI:** 10.1186/s41235-020-00269-8

**Published:** 2021-02-15

**Authors:** Megan H. Papesh, Michael C. Hout, Juan D. Guevara Pinto, Arryn Robbins, Alexis Lopez

**Affiliations:** 1 Department of Psychology, New Mexico State University, P.O. Box 30001/MSC 3452, Las Cruces, NM 88003 USA; 2 Rollins College, Winter Park, FL USA; 3 Carthage College, Kenosha, WI USA

**Keywords:** Visual search, Eye movements, Expertise

## Abstract

Domain-specific expertise changes the way people perceive, process, and remember information from that domain. This is often observed in visual domains involving skilled searches, such as athletics referees, or professional visual searchers (e.g., security and medical screeners). Although existing research has compared expert to novice performance in visual search, little work has directly documented how accumulating experiences change behavior. A longitudinal approach to studying visual search performance may permit a finer-grained understanding of experience-dependent changes in visual scanning, and the extent to which various cognitive processes are affected by experience. In this study, participants acquired experience by taking part in many experimental sessions over the course of an academic semester. Searchers looked for 20 categories of targets simultaneously (which appeared with unequal frequency), in displays with 0–3 targets present, while having their eye movements recorded. With experience, accuracy increased and response times decreased. Fixation probabilities and durations decreased with increasing experience, but saccade amplitudes and visual span increased. These findings suggest that the behavioral benefits endowed by expertise emerge from oculomotor behaviors that reflect enhanced reliance on memory to guide attention and the ability to process more of the visual field within individual fixations.

## Significance statement

We examined the development of expertise in a longitudinal visual search study, measuring how experience changes individual performance and gaze behaviors. Across 14 sessions throughout an academic semester, observers gained experience searching for items from 20 memorized categories among displays of 32 objects. Some displays contained no targets, and others contained up to three. With experience, observers became faster and more accurate. More importantly, their eye movements revealed that, relative to their average performance, experience allowed observers to rely more heavily on memory to identify objects efficiently and to process more of the visual field within each fixation. Although experts and novices may differ in many factors (e.g., interest, domain-specific education, etc.), our results confirm that the oculomotor behaviors associated with expert scanning are learned, rather than innate. These results carry implications for training and assessment in professional search domains and represent one of the only longitudinal studies to track how skill development influences gaze behaviors over time.

## Introduction

Across many domains and sensory modalities, expertise confers perceptual and cognitive benefits. Within visual domains, these benefits can include the abilities to efficiently extract relevant information from the environment, quickly process/perceive that information, and/or act on that information. For example, expert referees must direct attention to certain “contact zones” while monitoring for penalties (Spitz et al. 2016), quickly deciding whether one of many possible infractions has occurred before deciding to raise a card or throw a flag. These expert abilities typically result from accumulated experience rather than direct instruction, as verbalizing expert skills is difficult (e.g., Beilock and Carr 2001) and liable to impair skill execution (e.g., Flegal and Anderson 2008). Although performance failures in some domains are relatively inconsequential (e.g., sports refereeing), failure in other domains (e.g., radiology, airport baggage screening) can carry serious consequences. Moreover, experts in these consequential domains must contend with the fact that they are often scanning for something they rarely find. For example, in mammography, only 0.1% of medical images screened contain evidence of cancer (Krupinski 2010). In this study, we investigated how expertise develops across many sessions of practice in a laboratory search task, and how scanning, perceiving, and decision-making are affected by accumulating experience and target frequency.

Relative to novices, experts have been shown to execute fewer, or more systematic, eye movements while performing their expert tasks, including chess (Charness et al. 2001; Reingold and Charness 2005; Reingold et al. 2001), sports refereeing (e.g., Roca et al. 2013; Spitz et al. 2016), medical image screening (Drew et al. 2013; Kundel and La Follette 1972; Kundel et al. 2007; Nodine et al. 1996; Wood 1999), and baggage screening (e.g., Biggs et al. 2013; Biggs and Mitroff 2014), among others (see Brams et al. 2019, for a review and meta-analysis). For example, Reingold et al. (2001) gave a modified “check detection” task to novice, intermediate, and expert chess players: Players examined 3 × 3 subsections of chessboards to quickly determine whether the king was “in check.” (Three pieces were presented on the board, and none were presented in the central square.) Participants’ eye movements revealed that, relative to novice and intermediate players, experts were more likely to leave their gaze on the empty center of the board, indicating their check detection decision without moving their eyes. Moreover, when eye movements did occur, experts made fewer fixations than their less-skilled counterparts. This finding, that experts can perceive more of the board with fewer fixations, has since been replicated several times (Charness et al. 2001; Reingold and Charness 2005).

The ability to perceive more information with fewer eye movements may reflect alterations to experts’ functional viewing field (FVF).^1^ The FVF is the display area directly attended by observers, where items falling in foveal or parafoveal vision are processed with higher resolution (Sanders 1970). Items falling outside the FVF are processed peripherally, with lower resolution. Although visual processing is fundamentally limited by the distribution of photoreceptors in the retina, the FVF reflects the manner by which attention further affects central processing, such that task parameters and demands can alter the number of items processed in parallel from single fixations (Hulleman and Olivers 2017). For example, when targets are difficult to discriminate from distractors, the FVF “narrows” to reduce interference and facilitate individual item inspections. As target discriminability becomes easier, the FVF expands, allowing observers to inspect and reject multiple items from a single fixation. Because expert searchers rely on fewer eye movements to locate targets, they have been said to rely on a more “global” processing strategy (e.g., Manning et al. 2006), which a larger FVF facilitates.

An expanded FVF would be of limited utility without the ability to efficiently perceive the attended objects and avoid revisiting previously perceived scene regions. The role of memory, therefore, seems important to expert search performance. Across domains, researchers often find that experts exhibit superior ability to remember domain-specific material (see Gobet and Simon 1996; and Sala and Gobet 2017, for reviews). Chess experts, for example, can recall the names of various chess openings and rely on these memories to more efficiently perceive various arrangements (Chase and Simon 1973; Cooke et al. 1993; De Groot 1965). Within laboratory visual search tasks, observers’ performance is facilitated when searched-through scenes are familiar (Hout and Goldinger 2010, 2012; Võ and Wolfe 2012; Wolfe et al. 2011), allowing searchers to quickly avoid or reject distractors. Similarly, “inhibition of return” often acts as a mechanism that encourages orienting toward novel locations, allowing observers to avoid revisiting a previously inspected area (Klein 2000). Relative to novices, expert searchers often show more search systematicity, reflecting greater inhibition of return (Augustyniak and Tadeusiewicz 2006; Leong et al. 2007; Li et al. 2016).

After locating and perceiving a target, observers must decide whether to act on that information (e.g., note the presence of a radiological anomaly and throw a flag in football). Decision speed and accuracy often separate experts from novices, as experts have been found to be faster and/or more accurate than novices in sports (e.g., Alder et al. 2014; Casanova et al. 2013; Crespi et al. 2012; Del Campo et al. 2018; Hancock and Ste-Marie 2013; Piras et al. 2017; Schnyder et al. 2014; Williams et al. 1994; Williams and Davids 1998), radiology (e.g., Litchfield and Donovan 2016; Manning et al. 2006; Wood et al. 2013), and many other domains (see Brams et al. 2019). Beyond behavioral metrics of decision speed and accuracy, expertise effects can be observed in oculomotor behaviors, such as the duration observers spend examining specific items or areas (e.g., Nodine et al. 1996), or the latency between viewing a target and identifying it as meaningful. For example, experts typically spend more time examining regions with a high likelihood of containing a target and less time on regions containing distracting information (e.g., Gegenfurtner et al. 2011).

In a recent meta-analysis of search expertise, Brams et al. (2019) described many behavioral skills that differentiate experts from novices, and the cognitive and oculomotor variables that should reflect these differences across the different phases of individual search tasks. The earliest moments of a search task are characterized by pre-attentive processing, during which basic features (e.g., colors, orientations; Wolfe and Utochkin 2019) are registered. After the pre-attentive stage, selective attention works to guide attention in either a serial (Wolfe 2003; Woodman and Luck 2003) or parallel fashion (Hulleman and Olivers 2017; McElree and Carrasco 1999), depending on task parameters or theoretical framework. This guidance is based on knowledge of target-defining features (e.g., ketchup bottles are red) and also on learned information, such as scene regularities (e.g., ketchup is often found on countertops; Wolfe 2012; Wolfe et al. 1989). While searching through a scene, observers also engage memory processes: Working memory processes allow them to quickly retrieve relevant memories or experiences, which may be used to guide current perception, and long-term memory processes are used to commit scene details to memory or retrieve knowledge about scene regularities that can enhance guidance.

With accumulating expertise, any one or more of the phases of a typical search task may be facilitated. In some domains (e.g., medicine), experts are more likely to locate the target with their first fixation (Brams et al. 2019) and to have larger distances between successive fixations (Brams et al. 2020), both of which are consistent with an expanded FVF. In other domains (e.g., sports, air traffic control), experts show enhanced guidance and are likely to spend more time inspecting relevant scene regions (Brams et al. 2019). For example, expert chess players can more quickly identify the pieces from a legal, or structured, arrangement of chess pieces, relative to illegal, or unstructured, arrangements (Brockmole et al. 2008). Although the features of the individual chess pieces do not change across structured and unstructured arrangements, structured arrangements allow observers to rely on long-term knowledge to facilitate gaze behaviors and perception.

The literature on expertise in visual search is often restricted to between-groups designs: Experts in a specific field are compared to trainees or pure novices within that field. Although researchers are typically careful to equate various individual differences (e.g., visual acuity, education, age), one cannot control for innate or coincidental differences in visual skill that may encourage some people to self-select into professions that capitalize on that skill. In the present study, we used a relatively longitudinal approach to investigate expertise, such that each participant served as both a novice and, later, a skilled searcher with expert-level performance. Using this approach allowed us track changes to behavioral, cognitive, and oculomotor skills as expertise develops, rather than using between-group comparisons, which may be susceptible to individual-difference variations.

### The present investigation

In the present investigation, we monitored untrained searchers’ performance and oculomotor behaviors as they became adept at a laboratory visual search task designed to mimic some of the challenges faced by professional visual searchers, including the use of poorly specified and numerous potential targets, and targets which appeared with varying frequencies. Although many laboratory search tasks are guided by picture cues of targets, real-world search is often guided by categorical (i.e., word) cues, which impair, but do not preclude, attentional guidance (e.g., Schmidt and Zelinsky 2009). Additionally, as in many real-world search contexts, our search task involved multiple potential targets, drawn from many target categories (as in Cunningham and Wolfe 2014; Wolfe 2012). Although observers are able to search for many objects simultaneously, searching for multiple, relative to individual, items tends to make observers slower and less accurate (e.g., Menneer et al. 2007, 2009; Houtkamp and Roelfsema 2009; Schmidt et al. 2014; Stroud et al. 2012; Mestry et al. 2017). Moreover, using multiple-target search allowed us to measure decision processes, as observers did not know how many targets would be present in any given display. This ambiguity allows for meaningful search termination latencies.

Lastly, real-world search performance is often affected by the frequency with which observers encounter targets. In many applied domains, the most important targets appear relatively infrequently (e.g., a weapon in a carry-on bag). Despite their importance, such rare targets often go undetected, a phenomenon known as the low-prevalence effect (LPE; Wolfe et al. 2005). To be clear, the present study was not designed to investigate the LPE or mitigation strategies, both of which have been examined at length in other studies (e.g., Evans et al. 2013a, b; Godwin et al. 2015; Hout et al. 2015; Papesh et al. 2018; Peltier and Becker 2016; Walenchok et al. 2020; Wolfe et al. 2007; Wolfe and VanWert 2010). Indeed, the multiple-target nature of our paradigm made isolating frequency effects challenging, as observers could encounter multiple targets from the same-frequency category within a single trial. We manipulated how often observers encountered specific target categories to better reflect the conditions under which experts search for consequential and/or likely targets in applied domains.

The present investigation examined expertise development in a laboratory analog of a mixed prevalence, hybrid search task. By adopting a longitudinal approach, we were able to measure the behavioral (accuracy and response time), cognitive (decision time), and oculomotor (visits, dwell times, FVF, saccade amplitudes) measures that change as expertise develops.

## Method

### Participants

Thirteen unpaid research assistants from the laboratories directed by the first two authors volunteered to participate during their regularly scheduled laboratory hours (in lieu of data collection responsibilities). All participants were naïve to the purpose and design of the study, reported normal or corrected-to-normal vision (including color vision), and provided written informed consent. Participants completed a variable number of sessions (as many as their schedules would allow within a single semester), ranging from 6 to 23. In total, there were 192 experimental sessions recorded (14.77 sessions per participant, on average). To standardize (and maximize) the number of sessions in our analyses, we limited the sample to participants who completed at least 14 experimental sessions (*n* = 10). Only the first 14 sessions of these participants were included in analyses.

### Design

In each session, we manipulated trial type (0-, 1-, 2-, and 3-target present, in equal proportions) and category frequency, with categories appearing with variable frequency across all trials in an experimental session: least frequently (4 times), infrequently (8 times), frequently (16 times), and most frequently (32 times).

### Stimuli

**Fig. 1:**
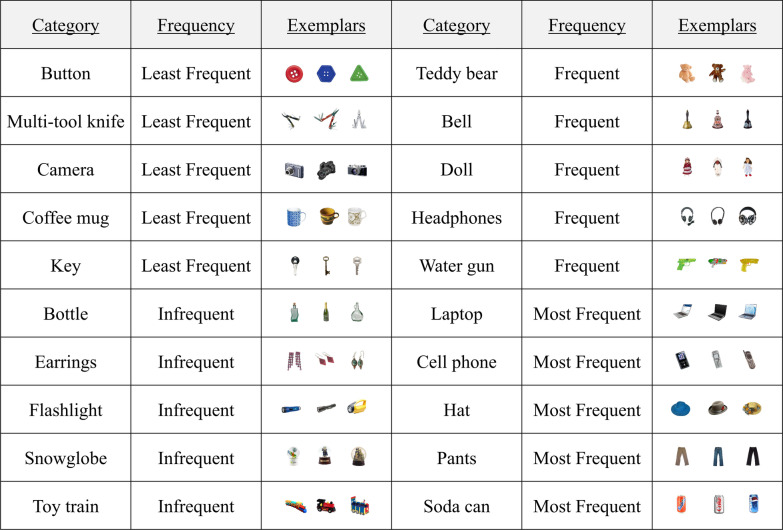
Target categories searched for by all participants, with corresponding frequency level. For each, three randomly chosen exemplars are displayed for demonstrative purposes, but each category was comprised of 16 possible exemplars in the experiment

All stimuli came from the “Massive Memory” database (Brady et al. 2008; Konkle et al. 2010) and were photographs of real-world objects from 240 distinct object categories, resized (maintaining original proportions) to a maximum of 2.5° of visual angle (horizontal or vertical) from a viewing distance of 55 cm. Images were no smaller than 2.0° of visual angle along either dimension. Each picture represented a single object or entity with no background. To populate the search arrays, targets were drawn from 20 categories (see Fig. 1), and distractors were drawn from 80 different categories. All image categories were made up of 16 exemplars (yielding 320 potential target images and 1280 potential distractor images). The 20 target categories were randomly and evenly divided across each level of category frequency, which was held constant across sessions. See Appendix Figs. 12 and 13 for a full list of distractor categories and sample exemplars.

### Apparatus

The experiment was controlled by EPrime vs.2 (Psychology Software Tools, Pittsburgh, PA) and conducted in two separate laboratories simultaneously. In one laboratory, the stimuli were presented on a 17″ CRT monitor with refresh rate of 75 Hz and screen resolution of 1920 × 1200, and in the other, the monitor was 24″ with a refresh rate of 60 Hz. Both laboratories used monocular eye tracking at 500 Hz using S-R Systems Eyelink 1000 or 1000 + trackers. Each participant only took part in the experiment at a single laboratory.

### Procedure

#### Eye tracking

Participants took part in the study individually. Participants used a chin rest during all trials and were calibrated (using a nine-point system) prior to each session. The chin rest was adjusted so each participant’s gaze landed centrally on the computer screen when the participant looked straight ahead. Calibration was accepted if the mean error was less than 0.5° of visual angle, with no error exceeding 1.0° of visual angle. Periodic recalibrations ensured accurate recording of gaze position throughout the experiment; recalibrations occurred at the beginning of each block and within blocks when necessary. (The option to recalibrate was provided at the start of each trial.) For analysis purposes, *interest areas* were defined as the smallest rectangular area that encompassed any given image. An eye movement was classified as a saccade when its distance exceeded 0.5° and its velocity reached 35°/s (or acceleration reached 9500°/s^2^). Viewing was binocular, but only the right eye was recorded.

#### Target category memorization and practice

During the first session, participants memorized the names of all target categories before performing any visual search trials; they performed two rounds of memorization and test. During memorization, participants viewed the full list of 20 target categories in a single alphabetized display, with a black box drawing their attention to each category name for 3 s before moving to the next category. After all categories were highlighted, participants completed a 40-trial old/new memory test (half old), using the keyboard to indicate whether the tested item was one that they were instructed to memorize. Accuracy was assessed after the second round of memorization and test. Participants could only continue to the visual search phase if they completed the memory test with 80% accuracy or better, else the memorization and test phase would be repeated. All participants performed the above criterion on the first try.

Following memorization, participants completed a practice block of 53 visual search trials (with 13 trials each for target-absent, 1-target, and 3-target trials; there were 14 2-target trials). In the practice block, each target category appeared with equal frequency (i.e., four times), so that frequency effects could only arise in experimental blocks.

#### Visual search

In each session, participants completed five 40-trial experimental blocks of visual search, with equal use of the four trial types (i.e., 0–3 target trials). At the start of each block (not trial), participants were reminded of the 20 target categories (using words, not pictures) for which they would be searching. When they were ready to begin, they pressed any key on the keyboard. To initiate each trial, participants clicked the mouse, after which a centrally presented, gaze-contingent fixation cross was shown. After participants fixated the cross for 500 ms, it disappeared and was replaced by the 32-object visual search array. Search arrays were constructed by dividing the entire screen into an invisible 6 × 6 grid, from which 32 (of 36 possible) locations were randomly chosen, with the provision that one cell within each screen quadrant remains empty. Precise target locations within each cell were jittered to ensure that a minimum of 1.5° visual angle separated items from each other and the edge of the display.

**Fig. 2:**
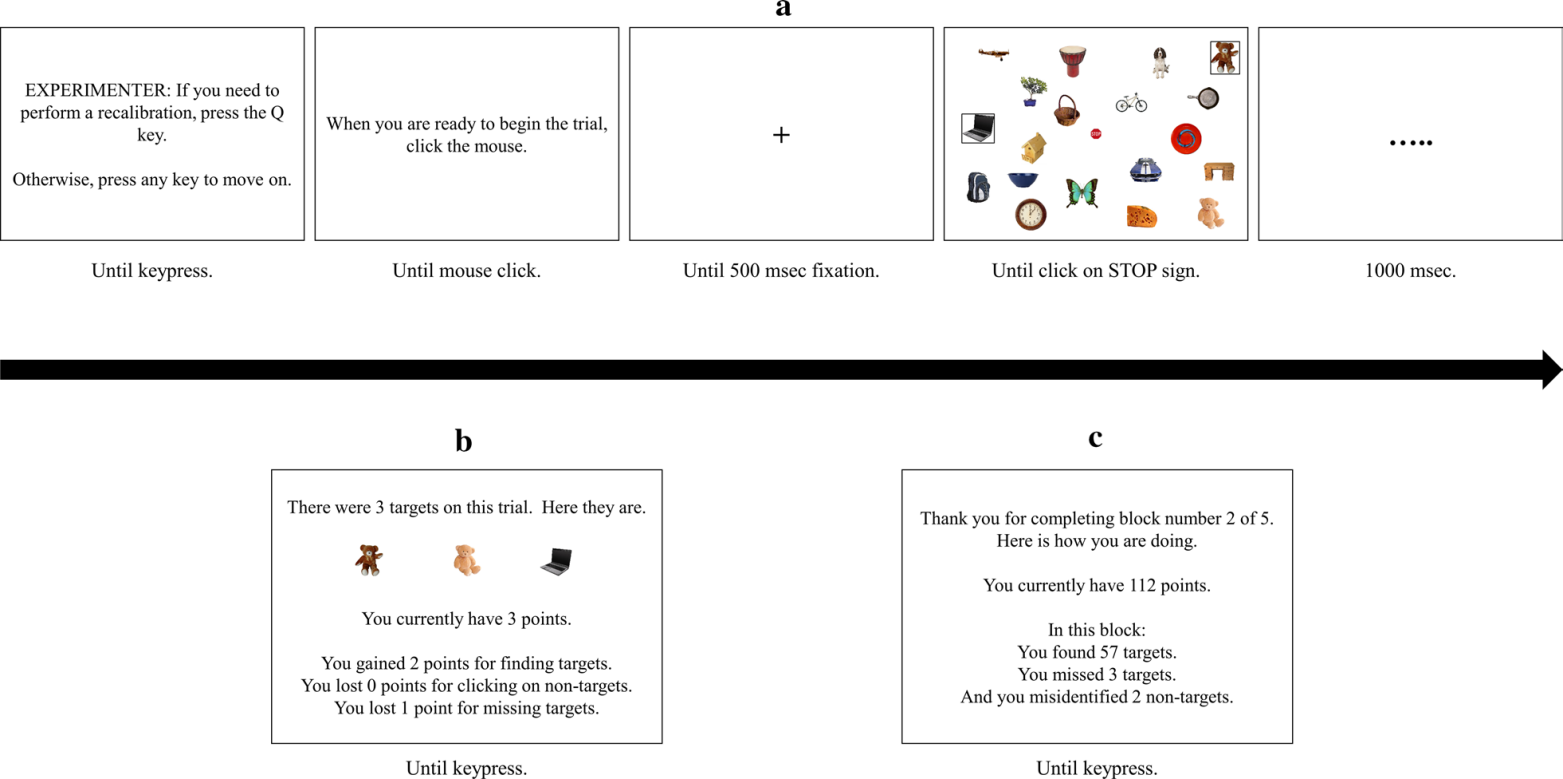
** a** Progression of events during a visual search trial. Borders drawn around objects indicate the participant selected them as targets. Note that the display is not drawn to scale, and 32 items were displayed on all trials. **b** The feedback that followed each practice trial. No feedback was present following experimental trials. **c** The feedback that followed each block of experimental trials

Within a single trial, targets could appear from across 20 categories, and multiple (non-identical) exemplars from a single category could also appear (however, no two distractors in any trial were from the same category). Participants indicated target selections by clicking on the pictures using the mouse. When pictures were selected, a black box was drawn around them to indicate that the computer detected the selection, but no indication was provided regarding whether the selected item was a target or distractor. Participants’ search was self-paced, and they terminated each trial by clicking on a “STOP” sign presented in the center of the display (see Fig. 2a for a sample trial progression). Participants’ goal was to gain as many “points” as possible over the experimental session. They gained one point for every “hit” and lost one point for every “miss” and every “false alarm.” Although a maximum of three targets appeared in any trial, participants were not told how many targets to expect on each trial and were not informed that target categories occurred with variable frequency.

#### Feedback

As shown in Fig. 2b, participants received trial-by-trial feedback during the practice block (which only occurred in the first session for each participant), so that they could adequately learn the target categories. After each practice trial, participants were shown the targets that appeared on that trial (if any), and the number of points they acquired. Points were reset to zero after the practice block. They were also told how many hits, false alarms, and misses occurred on that trial. During experimental blocks, feedback was only provided at the end of each block, at which point participants learned how many points they had acquired up to that point in that session (cumulatively across blocks; see Fig. 2c). They were also informed how many hits, misses, and false alarms they made. Information about specific categories and exemplars was not provided. This block-level feedback screen remained visible for as long as participants wished and therefore also served as a break between experimental blocks.

## Results

For each participant, performance on all dependent variables was baseline-corrected relative to that participant’s own mean performance across all sessions. This allowed us to examine the development of search expertise regardless of individual differences in performance, as reflected in the percentage change in performance over time (relative to the participant’s own mean performance). Thus, analyses presented in text were conducted on “change percentages” for each individual session relative to that participant’s own grand average across all sessions. Because of this scaling, only the main effect of session and interactions with it are interpretable. For other main effects, please see the supplemental analyses on raw data values in “Appendix 2.” For clarity and transparency, we plot group average data along with percent change data in the primary results, resulting in dual-axis figures: The left axis reflects percent change and the right axis reflects raw data. For all analyses, alpha level was set at 0.05, and multiple comparisons were subjected to Bonferroni corrections. Greenhouse–Geisser-corrected degrees of freedom are reported for any contrasts involving sphericity violations.

### Behavior: accuracy and response times (RTs)

**Fig. 3:**
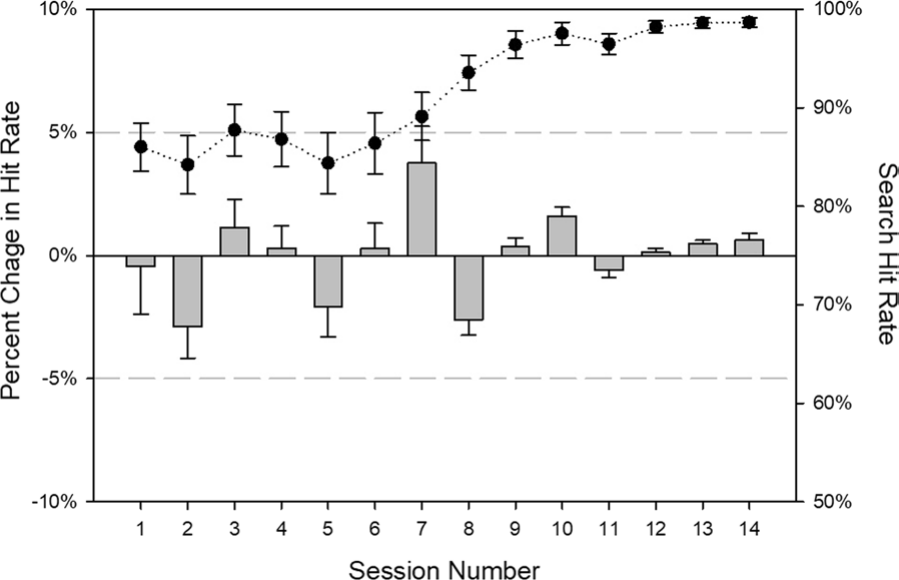
Average search hit rates (circles) and percent change in hit rates (bars) across sessions. Note that the percent change data are scaled relative to each participants’ grand average, but the raw values reflect group averages. Error bars reflect ± 1 standard error

Because the overarching hypotheses predict that expertise changes oculomotor measures, we treated analyses on behavioral measures as manipulation checks: Did accuracy and RT improve with accumulated experience? We examined search accuracy via hit rates (i.e., the number of targets correctly identified divided by the number present in the display)^2^ in a 14 (session) × 4 (target frequency: least frequent, infrequent, frequent, most frequent) RM ANOVA. We did not include the number of targets in the analysis because, within multiple-target trials, the targets could be drawn from one or more levels of target frequency, giving us uneven cells. The analysis revealed only a main effect of session, *F*(3.49, 31.41) = 2.95, *p* = 0.04; *η*_*p*_^2^ = 0.25. As shown in Fig. 3, searchers became better and more consistent at finding the target in the later sessions, relative to the earlier ones. These analyses suggest that observers developed the behavioral markers of expert-level performance and performed near ceiling by the later sessions. Analyses on raw hit rates confirmed no effect of target frequency^3^ (see Table 1; Fig. 14).

In our paradigm, expertise development can influence different aspects of the overall trial-level response time, including the latency to first target detection, the latency to click on all targets, and the overall time required for participants to terminate the trial. In the interest of brevity, we only report analyses on first target detection and search termination RTs in text, as these provide insight into expertise effects on search efficiency and quitting thresholds, respectively (full analyses can be found in “Appendix 2”).

**Fig. 4:**
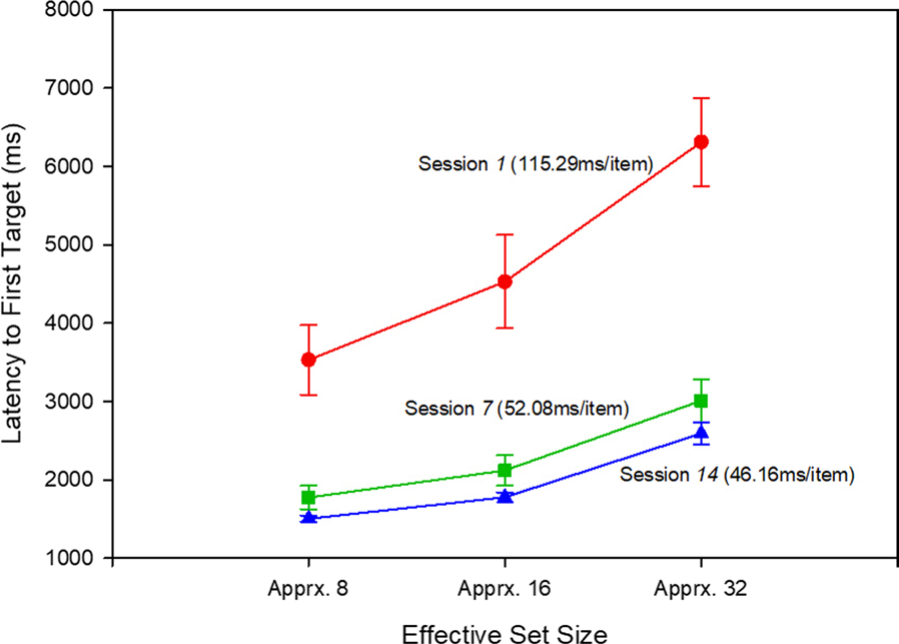
Average raw latency to the first target detection across effective set sizes 8, 16, and 32 for sessions 1, 7, and 14. Error bars reflect ± 1 standard error

Although we did not manipulate set size, the analysis examining the latency to first target detection offers a way to explore the effect of *effective* set size. Specifically, if observers must examine approximately half of the displayed objects before locating a target in a single-target trial, their effective set size in a 32-object display is 32. By virtue of already having scanned half of the objects, however, their effective set size for a two-target trial becomes 16, which then becomes (approximately) 8 for a three-target trial. We examined search efficiency in a 3 (effective set size) × 14 (session) RM ANOVA, which revealed a main effect of session, *F*(13, 117) = 39.10, *p* < 0.011, *η*_*p*_^2^ = 0.81, but no interaction, *p* = 0.53.^4^ For ease of interpretation, we plot raw search slopes in Fig. 4, showing search times as a function of effective set size in the first, middle, and final sessions. As shown in Fig. 4, search slopes were cut by more than half across the first and seventh sessions, after which they continued to decrease, albeit at a smaller rate, until session 14.

**Fig. 5:**
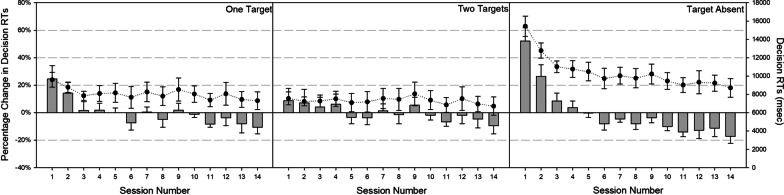
Decision RTs (circles) and percent change in RT (bars) across sessions for one-target (left panel), two-target (middle panel), and target-absent (right panel) trials. Error bars reflect ± 1 standard error

RTs may capture different phases of search, such as target identification or quitting decisions, each of which can be made more efficient by expertise development. Our paradigm also allowed us to isolate an additional cognitive process, search termination decisions. Because observers never knew how many targets would appear in any given trial, the latency between their final target detection and when they clicked “stop” in the 1- and 2-target trials can reveal insights into their quitting decisions (no more than 3 targets ever appeared in a trial, so quitting decisions in 3-target trials are less informative). Moreover, the latency to click the stop sign in target-absent trials may reflect a more global estimate of quitting thresholds (e.g., Chun and Wolfe 1996). We examined these percentage change click times in separate RM ANOVAs on session (both target-present and target-absent analyses) and number of targets (target-present trials only). Analysis on target-absent quitting RTs revealed a main effect of session, *F*(13, 117) = 11.10, *p* < 0.001, *η*_*p*_^2^ = 0.55. As shown in the right panel of Fig. 5, sessions 1 and 2 produced the slowest search termination decisions, which reliably differed from subsequent sessions (*p* < 0.05). By session 3, quitting times became stable and did not reliably differ. Analyses on target-present quitting RTs revealed a reliable interaction, *F*(13, 117) = 3.52, *p* = 0.01, *η*_*p*_^2^ = 0.28. As shown in the left panel of Fig. 5, this interaction was driven by relatively slower decision times for 1-target trials in the earliest sessions. As in the target-absent trials, decision speeds improved across the first three sessions, after which they became stable. Together, the target-absent and target-present data suggest that expertise may not necessarily speed-search termination decisions or affect quitting thresholds, instead reflecting a stable decision-making mechanism.

### Eye-tracking metrics

Because our paradigm was not designed to elicit frequency effects, we observed no effect of target frequency on accuracy, and we collapsed items into two discreet categories for eye-tracking analyses, targets and distractors. Frequency effects can, however, be found in several raw analyses reported in “Appendix 2”.^5^ Although eye-tracking affords many variables, we restricted our focus to visits (i.e., how often the eyes entered an interest area around targets or distractors), dwell times (i.e., how long visited items were viewed), FVF, and saccade amplitude.

#### Visits

Visits were defined as the number of times the eyes entered a given interest area divided by the total number of objects of that type (target or distractor). This calculation included zeroes, for rare instances in which a displayed object was not examined. Importantly, visits are consistent with, but not identical to, the number of fixations a given interest area received. For instance, if the eyes enter an interest area and commit two fixations before leaving the area, that would count as two fixations but only one visit. In this way, visits can be interpreted as the number of times each item was examined, irrespective of small corrective fixations that may have been committed within the interest area.

**Fig. 6:**
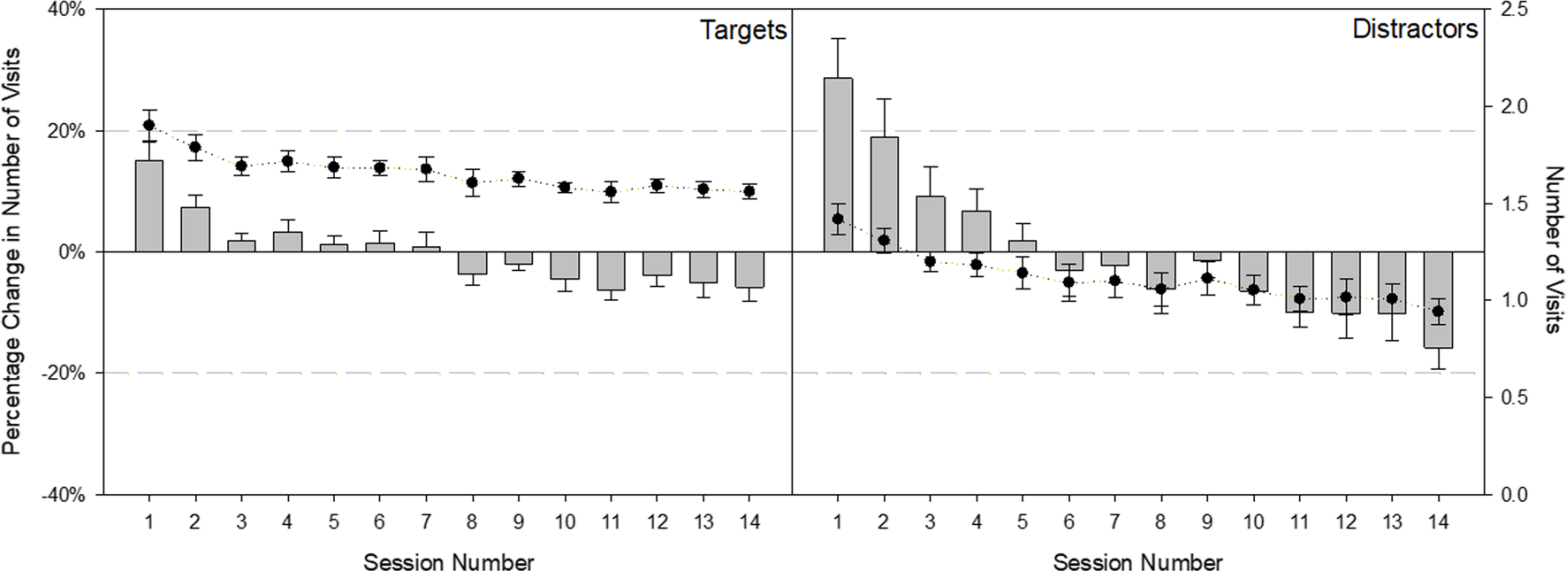
Average number of visits (circles) and percent change in average number of visits (bars) across sessions for targets (left graph) and distractors (right graph). Error bars reflect ± 1 standard error

As expertise develops, experts preferentially view target-relevant locations (Brams et al. 2019). To determine whether this was true in observers in the present investigation, we examined participants’ baseline-corrected average number of visits to target versus distractors in a 2 (Item Type: Target, Distractor) × 14 (session) RM ANOVA. There was a main effect of session, *F*(13, 117) = 9.17, *p* < 0.01, *η*_*p*_^2^ = 0.51, which revealed that the probability of fixating items decreased with increasing experience across sessions. This main effect, however, was qualified by a reliable interaction, *F*(13, 117) = 4.31, *p* < 0.01, *η*_*p*_^2^ = 0.32. As shown by the bar graphs in Fig. 6, which represent how participants’ performance changed relative to their own grand average, the probability of fixating on targets and distractors changed across sessions. Simple main effects confirm that distractors received relatively more fixations than targets in sessions 1 and 2 (*F*_S1 _= 10.38,* p* = 0.01; *F*_S2_ = 6.46, *p* = 0.03) but that targets received relatively more fixations than distractors in session 14 (*F*_S14_ = 14.47, *p* = 0.01). Although targets are obviously more likely to be viewed than distractors in the raw data (circles in Fig. 6), the percent change data reveal how these viewing preferences change across sessions.

#### Dwell times

**Fig. 7:**
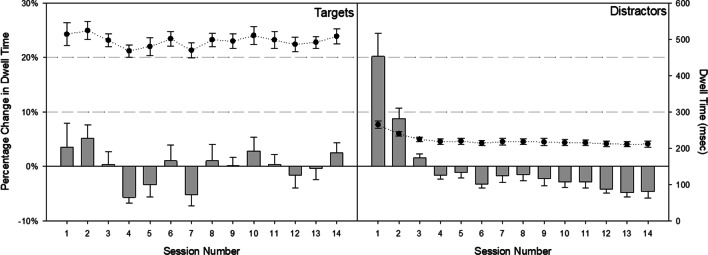
Average dwell time (circles) and percent change average dwell time (bars) across sessions for targets (left graph) and distractors (right graph). Error bars reflect ± 1 standard error

For each item visited, we calculated the average amount of time participants spent on each visit as a measure of object identification. Because experts are better able to rely on memory processes during search (e.g., Brockmole et al. 2008), dwell times should change as observers accrue experience: Distractors should be viewed for less time, relative to targets, although all dwell times should generally decrease (reflecting enhanced object identification abilities). To evaluate these predictions, we examined baseline-corrected average dwell times in a 2 (Item Type) × 14 (session) RM ANOVA.^6^ We observed a main effect of session, *F*(13, 117) = 7.90, *p* < 0.01, *η*_*p*_^2^ = 0.47: Relative to their grand average dwell times, participants’ dwell times decreased across sessions (Fig. 7). We also, however, observed a reliable interaction, *F*(13, 117) = 4.12, *p* < 0.01, *η*_*p*_^2^ = 0.31. Simple effect tests confirm that the interaction was in the predicted direction: In early sessions, distractors were looked at longer than average, relative to targets (*F*_S1_ = 9.18*, p* = 0.01; *F*_S4_ = 12.99, *p* = 0.01), but by session 14, this relationship flipped (*F*_S14_ = 9.60*, p* = 0.01), which may reflect a change in quitting threshold.

#### Functional viewing field (FVF)

**Fig. 8:**
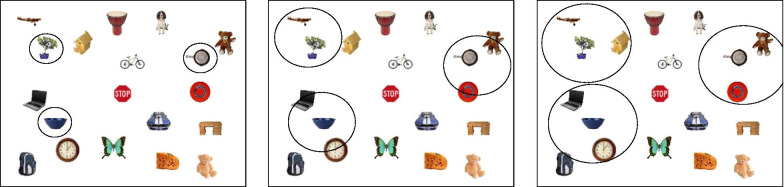
Hypothetical 18-item display for a single-target search. To calculate FVF, circles are drawn around each fixation point, beginning with a radius of 1 degree (left panel). The radius of the circle is gradually increased until 50% of the items fall within one of the circles (right panel). *Note* The figure is not drawn to scale

Relative to novices, experts are better able to quickly direct attention to relevant screen locations while ignoring distracting or irrelevant information (Brams et al. 2019, 2020). The extended visual span implied by such results may arise from task-specific experience or it may be related to self-selection biases (e.g., those with an extended visual span may be drawn to professions in which that ability would be useful). To examine whether visual span extends as expertise develops, we examined changes in FVF. To calculate initial FVF, we used the method described by Young and Hulleman (2013),^7^ which involves first drawing invisible circles with 1º of visual angle radii centered on all fixations (e.g., Fig. 8, left panel). The radius is then increased by 1^◦^ until a given proportion of objects fall within one of the circles (Fig. 8, middle and right panels), with each object only counted once across all fixations (i.e., if an object falls within the circle drawn around more than one fixation, that object is still only counted once). The formula for calculating the critical proportion is (set size + 1)/(number of targets + 1). For a set size of 32, that means that 16.5, 11, and 8.25 objects must be encircled on 1-, 2-, and 3-target trials, respectively. Because an observer cannot fixate a partial object, the criterion for 1-, 2-, and 3-target trials was rounded up to 17, 11, and 9 objects, respectively. This corresponds to just more than 50% of the objects on 1-target trials and comparatively less when multiple targets are present in the display (34.38% and 25.78% of objects for 2- and 3-target trials, respectively). For example, in the hypothetical 18-item display in Fig. 8, the critical proportion for a single-target trial would be 50% (9 items). The FVF is the size of the fixation radii that encompasses 9 items (Fig. 8, right panel).

**Fig. 9:**
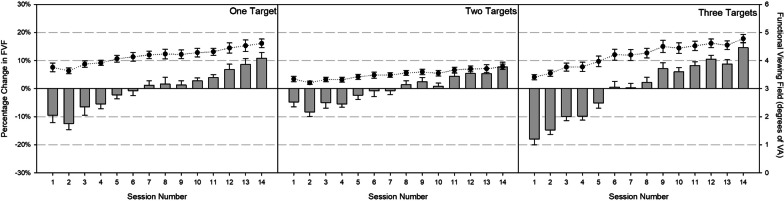
Functional viewing field (circles) and percent change in functional viewing (bars) across sessions for one- (left graph), two- (middle graph), and three-target (right graph) trials. Error bars reflect ± 1 standard error

To determine whether experience and the number of targets in the display influence observers’ initial FVF sizes, we examined FVF in a 3 (number of targets) × 14 (session) RM ANOVA. This analysis confirmed a reliable effect of session, *F*(4.49, 40.18) = 22.18, *p* < 0.001; *η*_*p*_^2^ = 0.71, and a reliable interaction between session and number of targets, *F*(4.47, 40.25) = 5.6, *p* = 0.001; *η*_*p*_^2^ = 0.38. Observers’ ability to process multiple objects from a single fixation increased with experience, which was reflected in a percentage increase in FVF size across sessions. As shown in Fig. 9, the interaction was characterized by greater session-by-session stability for trials including two targets relative to trials including one or three targets.

**Fig. 10:**
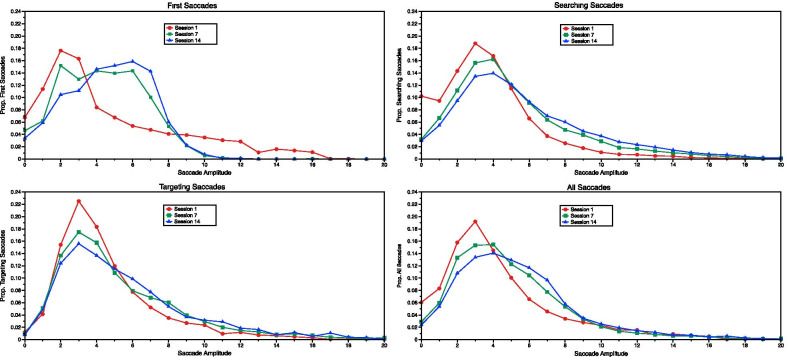
Proportion of first (upper left), searching (upper right), targeting (lower left), and overall (lower right) saccades by their amplitude in sessions 1 (red circles), 7 (green squares), and 14 (blue triangles)

Although the FVF changed with accumulated experience, the measure has not been without criticism (e.g., Kristjánsson et al. 2017), and alternative estimation procedures exist. One alternative to FVF calculations involves measuring saccade amplitudes: With a larger visual span, observers are able to execute higher amplitude saccades, covering a greater portion of the viewing area. The benefit of measuring saccade amplitudes lies in its within-trial flexibility: Whereas our calculated FVF measure assumes that the FVF remains stable throughout the trial, saccade amplitudes can be measured throughout the duration of trials, allowing saccades to be labeled based on search phase. In this way, we identified three trial periods during which FVF might change with the development of expertise^8^: (1) At the initiation of the trial, (2) during the active searching portion of the trial, and (3) when a fixation is first directed to a target. The amplitude of the first saccade off the central fixation cross captures the FVF at the beginning of the trial; larger first saccade amplitudes imply a greater pre-attentive visual span. We operationalized searching saccades as those occurring between 2 and 9 saccades prior to the one directing attention to the target, and targeting saccades as the final saccade directing attention to the target. Figure 10 shows the proportion of saccade amplitudes for each of these saccades, separately and collapsed together, as a function of accumulating experience (sessions 1, 7, and 14). To determine whether the distributions shown in each panel of Fig. 10 differed, we conducted a series of Kolmogorov–Smirnov (K–S) tests (Holliday 2017), comparing the saccade distributions for the first, middle, and final sessions within each saccade category. Despite the apparent distributional shift in the first saccade amplitudes (Fig. 10, upper left panel), none of the K–S tests revealed any reliable differences, all *p*s > 0.3.

**Fig. 11:**
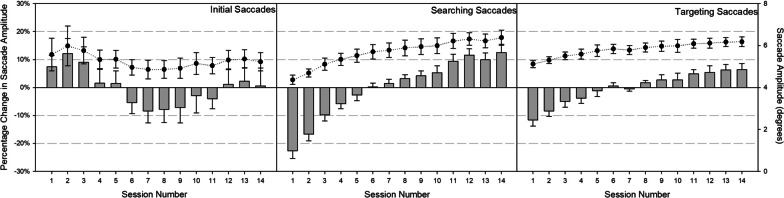
Raw saccade amplitudes (circles) and percent change in saccade amplitudes (bars) across sessions for initial saccades (left panel), searching saccades (middle panel), and targeting saccades (right panel). Error bars reflect ± 1 standard error

**Fig. 14:**
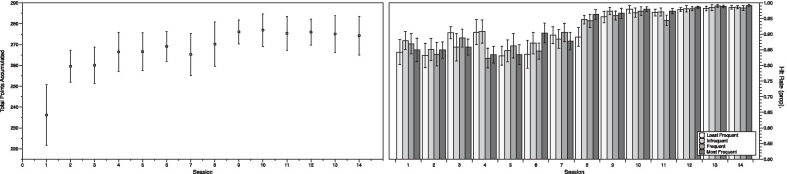
Total points accumulated across sessions (left panel) and hit rates across sessions as a function of target frequency (right). Error bars are standard error

Although the distributions of the saccade amplitudes in each panel of Fig. 10 did not change with experience, we analyzed the percentage change to participants’ mean saccade amplitudes for their first, searching, and targeting saccades in separate RM ANOVAs on all 14 sessions.^9^ Consistent with the K–S tests on to the first, middle, and final sessions, analyses on the first saccade amplitude revealed no effect of session, *F*(13, 117) = 0.67, *p* = 0.79; *η*_*p*_^2^ = 0.07. Both searching and targeting saccade amplitudes reliably changed across sessions, F_S_(13, 117) = 22.00, *p* < 0.001; *η*_*p*_^2^ = 0.71; *F*_T_(13, 117) = 8.10, *p* < 0.001; *η*_*p*_^2^ = 0.47. As shown in Fig. 11, searching and targeting saccade amplitudes increased with increasing experience, reflecting a gradual expansion of the FVF during search phases following the initial saccade off the fixation cross.

## Discussion

The present study examined behavioral and oculomotor measures of expertise development in a multiple-target hybrid search task conducted longitudinally over the course of a single semester. With practice, observers became faster and more accurate at searching for categorically defined targets, as would be expected in many real-life domains (e.g., sports, medicine, security screening).^10^ The development of this expertise also changed the way that viewers examined the visual field: With growing experience, observers needed to visit objects less frequently, they were less likely to view distractors, they viewed objects for shorter durations, and they showed evidence of an expanding visual span, particularly when searching for and locating items. Although abundant research has compared novice to expert search behaviors (e.g., Bilalić et al. 2011; Brams et al. 2020; Godwin et al. 2015; Reingold et al. 2001; Reingold and Sheridan 2011; Van Meeuwen et al. 2014, among many others), the present study adopted a within-subjects design to reveal how experience modifies scanning behaviors while eliminating the possibility of innate between-group differences in skill or interest level.

Changes in gaze behavior underlie the perceptual-cognitive benefits enjoyed by experts over novices (Brams et al. 2019), and the present research suggests that these changes are learned, rather than inherent individual differences. Brams et al. (2019) conducted a meta-analysis across three domains of visual search expertise, including sports (e.g., refereeing), medicine (e.g., radiology), and other areas (e.g., air traffic control, chess). Across domains, experts located targets more quickly, preferentially examined target-relevant scene regions, decreased viewing times, and increased saccade amplitudes. Our experiment replicates and adds to this growing literature, showing that these changes do not exclusively separate groups of experts from groups of novices. Instead, these changes occur gradually as a novice becomes an expert. In the present investigation, observers gradually decreased their dwell times and visited across sessions, and their eye movements revealed experience-dependent increases in saccade amplitudes and visual spans (via FVF).

Understanding how search skills become refined with experience may inform the development of training protocols or assessments. In a recent training study, Sha et al. (2020) found that novices’ ability to spot tumors in chest radiographs improved across four days of training, but this ability only transferred to novel (untrained) radiographs when the training images included both the tumor and some background. Training with images depicting only the tumor or only the background yielded improvement restricted to trained images. That observers need both local properties (the tumor) and its contrast with surrounding regions to best perceptually learn suggests that restricted viewing conditions do not benefit learning. They also do not seem to benefit performance at testing. Although presenting observers with limited viewing windows decreases overall perceptual load, it has a negative impact on search performance, particularly in conditions that encourage larger functional viewing fields (e.g., Young and Hulleman 2013). In the present study, and many others (Chin et al. 2018; Drew et al. 2013; Evans et al. 2013a, b; Evans et al. 2016; Nodine et al. 1999), experience confers the ability to utilize more of the visual display at any one time, suggesting that training or assessment methods that restrict observers’ views, or highlight small to-be-searched regions, will be of limited utility. Indeed, this may be one reason why computer-assisted detection methods often fail to improve target detection (e.g., Drew et al. 2020; Fenton et al. 2011; Philpotts 2009).

If image restriction techniques cannot be used to streamline the development of expertise, what can be done? Kramer et al. (2019) discussed three aspects of search performance that can be trained in professional searchers: (1) Efficient use of the technology, (2) target and distractor recognition, and (3) search strategies. Although technological training is important, it is beyond the scope of the present research. Instead, our results potentially impact the remaining two aspects of performance. Clearly, perceptual learning is important for target and distractor recognition (Sha et al. 2020), and our results confirm that searchers gradually learn to recognize both targets and distractors, visiting them less often as experience accumulates. Although we did not give observers search strategy instructions, the saccade amplitude and FVF analyses suggest that search strategies may have changed with increased experience, opening up the possibility that this behavior can be directly trained or measured.

Although training search strategies generally focus on teaching observers where to look or how to minimize decision errors (Kramer et al. 2019), measuring search strategies may hold promise for identifying expert-level performance. For example, with growing perceptual experience and span (Brams et al. 2020) in a particular domain, experts may begin to rely more on passive cognitive strategies than active ones. In passive search, observers make fewer, but more sweeping, eye movements, allowing targets to “pop out” rather than exerting cognitive control over attentional guidance (Madrid and Hout 2019; Smilek et al. 2006; Watson et al. 2010). Whether experts adopt a passive strategy, or merely have eye movement characteristics consistent with passive search, remains an open question.

By monitoring observers’ eye movements as experience accumulated, we were able to estimate changes in each phase of visual search, from pre-attentive processing through guidance and, ultimately, object identification and search termination. During pre-attentive processing, observers’ merely register basic features, such as color or line orientation (Wolfe and Utochkin 2019). Should this phase of search be facilitated by growing expertise, we would have expected first saccade amplitudes to increase, reflecting observers’ ability to pre-attentively take in more of the visual display. We did not observe this. Instead, we found that subsequent phases of search were facilitated by expertise. With experience, observers’ searching and targeting saccades gradually became longer, revealing two novel insights into search performance: (1) FVF size changes within search trials and (2) FVF size changes across search trials. Although the FVF has been shown to change with task demands (e.g., Hulleman and Olivers 2017) and across groups of experts and novices (e.g., Brams et al. 2020), our research shows that it also changes within individuals as a function of experience, both within-individual search trials and more globally, as experience develops. These changes to the FVF may have also permitted observers to direct attention to distracting objects less often, making search more efficient. In addition to lengthening the searching and targeting saccade amplitudes, experience also refined the final phases of visual search: object identification and search termination. Both object identification and search termination became faster with experience, which is consistent with expertise effects across many domains (see Brams et al. 2019) and with prior research showing the importance of memory for visual search (e.g., Brockmole et al. 2008).

In sum, we found that, as searchers gained expertise, they became better able to direct their attention to relevant locations, reflecting increased reliance on memory and/or an extended visual span. This is notable, given that searchers looked for twenty categories simultaneously among thousands of different distractor pictures, with no ability to predict which particular target features would be useful on any given trial. The present study revealed that expertise in visual search may refine multiple attentional, perceptual, and oculomotor skills, including the allocation and restriction of attention, object identification, and the speed and amplitude of saccadic eye movements. This investigation also uncovered new questions about the development of expertise in visual search, and whether these gaze behaviors are amenable to training. For example, future work will be needed to determine the extent to which expertise-induced changes in visual span can be affected by training or other manipulations (e.g., global/local bias inductions), and whether these changes mitigate the LPE. Moreover, experience-based increases in visual span have important implications for theories of visual search, which may need to incorporate future model adjustments to address this modifiable parameter.

## Figures and Tables

**Fig. 12:**
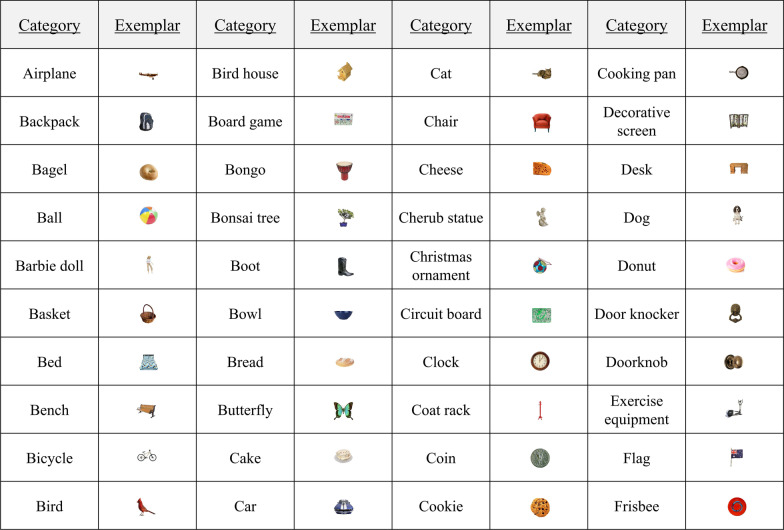
Forty (of 80) distractor categories encountered by participants (see also Fig. 13). For each, a single representative exemplar is displayed for demonstrative purposes, but each category was comprised of 16 possible exemplars in the experiment. Participants were not informed of the category names for distractors

**Fig. 13:**
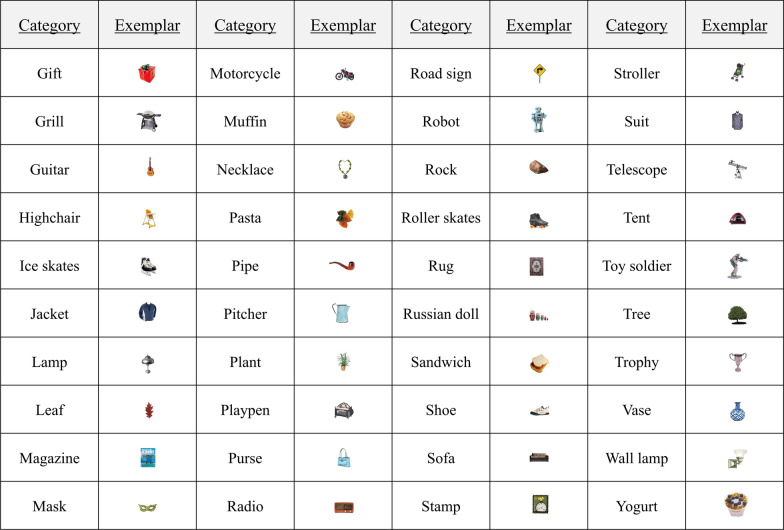
Forty (of 80) distractor categories encountered by participants (see also Fig. 12). For each, a single representative exemplar is displayed for demonstrative purposes, but each category was comprised of 16 possible exemplars in the experiment. Participants were not informed of the category names for distractors

**Fig. 15:**
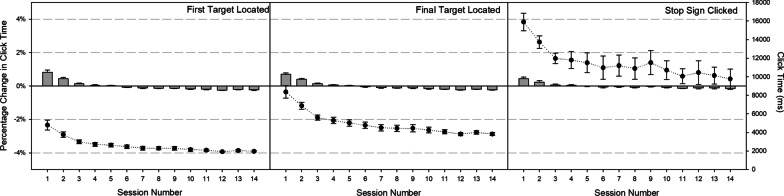
Average raw (circles) and percent change (bars) click time RT across sessions for locating the first target (left panel), the final target (middle panel), and the trial-terminating stop sign (right panel). Error bars reflect ± 1 standard error

**Fig. 16:**
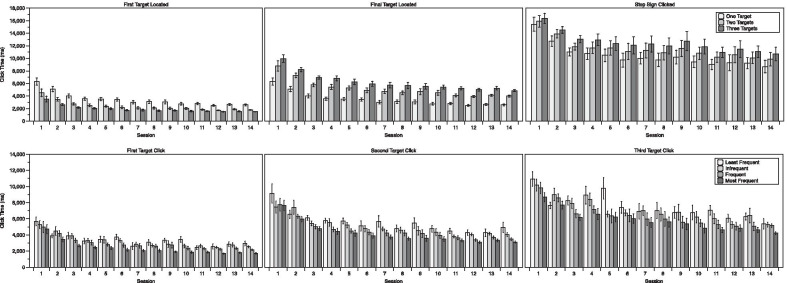
Top: Average click times (ms) for locating the first target (left panel), final target (middle panel), and the stop sign to end the trial (right panel) as a function of the number of targets present in the display. Bottom: Average time (ms) to click on the first (left panel), second (middle panel), and third (right panel) targets in multiple-target trials as a function of target frequency. Error bars are standard error

## Data Availability

The datasets generated and analyzed during the current study are available in the OSF repository, https://osf.io/emhrv/?view_only=c740e56448dc45a9acb051238ac20fb7.

## Appendix 1

See Figs. 12 and 13.

## Appendix 2

See Tables

**Table 1:** Results of analyses on raw value accuracy measures

Dependent variable	Effect	df	*F*	*p*	*η*_*p*_^2^
Points acquired	Session*	2.72, 24.5	8.41	< 0.001	0.483
Trial-level hit rate	Session (S)*	3.49, 31.43	18.65	< 0.001	0.674
	Target frequency (TF)	1.93, 17.23	0.925	0.412	0.093
	S × TF	4.49, 40.41	1.673	0.169	0.157

^*^Significant effects

1,

**Table 2:** Results of analyses on multiple percentage change RT measures

Dependent variable	Effect	*df*	*F*	*p*	*η*_*p*_^2^
Latency to first target	Number of targets (NT)	2, 18	0.01	0.987	0.001
	Session (S)*	13, 117	39.10	< 0.001	0.81
	NT × S	26, 234	0.95	0.534	0.01
Latency to all targets	Number of targets (NT)	2, 18	2.18	0.142	0.19
	Session (S)*	13, 117	57.19	< 0.001	0.86
	NT × S*	26, 234	2.86	0.022	0.24
Trial termination	Number of targets (NT)	2, 18	1.57	0.235	0.15
	Session (S)*	13, 234	9.71	< 0.001	0.52
	NT × S*	26, 234	3.29	0.011	0.27
First target click	Target frequency (TF)	3, 27	1.80	0.183	0.17
	Session (S)*	13, 117	38.41	< 0.001	0.81
	TF × S	39, 351	1.74	0.124	0.16
Second target click	Target frequency (TF)	3, 27	0.872	0.433	0.09
	Session (S)*	13, 117	34.55	< 0.001	0.79
	TF × S	39, 351	1.72	0.125	0.16
Third target click	Target frequency (TF)	3, 27	1.88	0.222	0.27
	Session (S)*	13, 117	25.37	< 0.001	0.84
	TF × S	39, 351	1.44	0.252	0.22

^*^Significant effects

2,

**Table 3:** Results of analyses on raw value RT measures

Dependent variable	Effect	df	*F*	*p*	*η*_*p*_^2^
Latency to first target	Number of targets (NT)*	2, 18	152.55	< 0.001	.94
	Session (S)*	13, 117	25.93	< 0.001	0.74
	NT × S*	26, 234	9.32	< 0.001	0.51
Latency to all targets	Number of targets (NT)*	2, 18	365.03	< 0.001	0.976
	Session (S)*	13, 117	40.1	< 0.001	0.817
	NT × S*	26, 234	2.68	0.03	0.229
Latency to trial termination	Number of targets (NT)*	2, 18	26.87	< 0.001	0.749
	Session (S)*	13, 117	7.61	0.002	0.458
	NT × S	26, 234	1.33	0.279	0.128
First target click	Target frequency (TF)*	3, 27	46.93	< 0.001	0.84
	Session (S)*	13, 117	25.37	< 0.001	0.74
	TF × S	39, 351	0.96	0.457	0.09
Second target click	Target frequency (TF)*	3, 27	40.2	< 0.001	0.82
	Session (S)*	13, 117	22.59	< 0.001	0.72
	TF × S	39, 351	1.03	0.416	0.10
Third target click	Target frequency (TF)*	3, 27	23.14	< 0.001	0.82
	Session (S)*	13, 117	21.37	< 0.001	0.81
	TF × S	39, 351	1.43	0.258	0.22
Target-absent trial termination	Session (S)	3, 117	8.50	< 0.001	0.49

^*^Significant effects

3,

**Table 4:** Results of analyses on raw value eye-tracking measures

Dependent variable	Effect	df	*F*	*p*	*η*_*p*_^2^
Visits	Item type (IT)*	1, 9	59.58	< 0.001	0.87
	Session (S)*	13, 117	8.60	< 0.001	0.49
	IT × S	13, 117	1.13	0.339	0.11
Dwell times	Item type (IT)*	1, 9	211.69	< 0.001	0.96
	Session (S)*	13, 117	3.70	< 0.001	0.29
	IT × S	13, 117	1.48	0.134	0.14
Functional viewing field	Number of targets (NT)*	1.18, 10.6	18.73	0.001	0.68
	Session (S)*	4.41, 39.66	23.54	< 0.001	0.72
	NT × S*	3.69, 33.24	6.47	0.001	0.42

^*^Significant effects

4 and

**Table 5:** Results of analyses on raw value saccade amplitudes

Saccade(s)	Effect	df	*F*	*p*	*η*_*p*_^2^
First	Session	3, 117	0.64	0.82	0.07
Searching	Session*	3, 117	20.3	< 0.001	0.69
Targeting	Session*	3, 117	8.13	< 0.001	0.47

^*^Significant effects

5 and Figs. 14, 15 and 16.

## Abbreviations

FVF: Functional viewing field
LPE: Low-prevalence effect
RT: Response time
RM ANOVA: Repeated-measures analysis of variance

## Footnotes

1 Similar concepts have been articulated, albeit with different names, such as the useful field of view (Ball et al., 1988) or perceptual span (O’Regan et al. 1983).

2 Analyses were not conducted on false alarm rates due to the low number of false alarms (0.5–3.5%) per session.

3 Although frequency effects are often observed in the literature, we did not predict them in our study because multiple targets from a given frequency category could appear within a trial. We observed frequency effects in some of the raw data analyses presented in Appendix (prevalence effects, per se, were precluded by the task-wide high prevalence of targets, of course).

4 Note that, because we analyzed percentage change relative to the participants’ overall mean, a main effect of effective set size is not possible in this analysis. Please see the raw analyses in “Appendix 2” for such effects.

5 Although we do not report raw analyses in text, it is worth nothing that a 4 (target frequency) × 14 (session) RM ANOVA on average raw dwell times revealed the predicted main effect of target frequency, *F*(1.73, 15.58) = 49.61, *p* < .01, *η*_*p*_^*2*^ = .85. As frequency increased, average dwell times decreased. Target frequency did not affect any other oculomotor measures.

6 As shown by the circles in Fig. 7, targets received longer dwell times than distractors, but this is an artifact of the design: Participants were required to fixate, and then click on, targets, thereby encouraging longer dwell times. For this reason, we emphasize the baseline-corrected data (bar graphs).

7 We are grateful to Johan Hulleman (personal communication) for assistance with these calculations.

8 We are grateful to Jeremy Wolfe for this suggestion.

9 A complementary analysis conducted on median saccade amplitudes yielded identical effects.

10 It is worth noting, however, that observers in our study did not begin as true novices (i.e., performing at or below chance prior to training), as might be expected in some professional domains.
